# Wind tunnel‐based testing of a photoelectrochemical oxidative filter‐based air purification unit in coronavirus and influenza aerosol removal and inactivation

**DOI:** 10.1111/ina.12847

**Published:** 2021-05-07

**Authors:** Yuechen Qiao, My Yang, Ian A. Marabella, Devin A.J. McGee, Bernard A. Olson, Montserrat Torremorell, Christopher J. Hogan

**Affiliations:** ^1^ Department of Mechanical Engineering College of Science and Engineering University of Minnesota Minneapolis MN USA; ^2^ Department of Veterinary Population Medicine College of Veterinary Medicine University of Minnesota Saint Paul MN USA

**Keywords:** air purifier, coronavirus, filtration, influenza, photoelectrochemical oxidation, virus aerosols, wind tunnel test

## Abstract

Recirculating air purification technologies are employed as potential means of reducing exposure to aerosol particles and airborne viruses. Toward improved testing of recirculating air purification units, we developed and applied a medium‐scale single‐pass wind tunnel test to examine the size‐dependent collection of particles and the collection and inactivation of viable bovine coronavirus (BCoV, a betacoronavirus), porcine respiratory coronavirus (PRCV, an alphacoronavirus), and influenza A virus (IAV), by a commercial air purification unit. The tested unit, the Molekule Air Mini, incorporates a MERV 16 filter as well as a photoelectrochemical oxidating layer. It was found to have a collection efficiency above 95.8% for all tested particle diameters and flow rates, with collection efficiencies above 99% for supermicrometer particles with the minimum collection efficiency for particles smaller than 100 nm. For all three tested viruses, the physical tracer‐based log reduction was near 2.0 (99% removal). Conversely, the viable virus log reductions were found to be near 4.0 for IAV, 3.0 for BCoV, and 2.5 for PRCV, suggesting additional inactivation in a virus family‐ and genus‐specific manner. In total, this work describes a suite of test methods which can be used to rigorously evaluate the efficacy of recirculating air purification technologies.

**Practical Implications**

- The standard method of evaluating the performance of recirculating air purification units is via inference of the clean air delivery rate (CADR); this is usually performed in a test of particle concentration decay in a room. While relatively simple to implement, such tests require correction for particle deposition within the room and are influenced by imperfect mixing.
- In adapting wind tunnel testing to recirculating air purification units, we are able to accurately determine their size‐dependent collection efficiencies, and their clean air delivery rates (product of collection efficiencies and operating flow rates).
- By placing the wind tunnel within a biosafety level II facility we are able to directly examine the efficacy in collecting and inactivating airborne viruses, with collection and inactivation distinguishable from one another via use of both virus titration and reverse transcription quantitative polymerase chain reaction assays.

## 1 INTRODUCTION

The coronavirus disease 2019 (COVID‐19) pandemic has led to considerable public attention on methods to mitigate viruses that transmit through aerosols. There is evidence that aerosol‐based transmission, either via direct inhalation of infectious particles, or indirect via deposition of particles and subsequent infection from surface transfer, is an important infection route for severe acute respiratory syndrome coronavirus type 2 (SARS‐CoV‐2), the coronavirus responsible for COVID‐19.^1^, ^2^, ^3^, ^4^, ^5^, ^6^, ^7^ Sufficiently small aerosol particles (distinguished in the medical community from “droplets,” ie larger entities which gravitationally or inertially deposit seconds after release^8^, ^9^), once emitted, have longer lifetimes in indoor spaces unless they are actively removed via ventilation. While the most direct method of addressing aerosol clearance is to modify heating ventilation air conditioning (HVAC) systems to operate at higher air change rates, such modifications are often costly, particularly in older residential and commercial buildings, which can operate without or with limited forced air heating and cooling systems. A possible alternative to effectively increase ventilation rates, or to locally reduce particle and airborne virus concentrations in an indoor space, is use of recirculating air purification units, which can incorporate filters, UV‐light, electrostatic precipitation, and photoelectrochemical oxidation technologies in order to collect, react, or inactivate pollutants and pathogens in indoor aerosols.

At the time of writing this manuscript, there is a tremendous number of recirculating air purification technologies, largely filter based, on the consumer market. However, most of these commercial systems have only been coarsely tested in terms of their abilities to mitigate either non‐infectious or infectious aerosol exposure. It is not common to evaluate the size‐dependent collection efficiencies of these devices, as the Association of Home Appliance Manufacturers (AHAM) testing standard AC–1–2015 only describes testing with particles in three broad classes without size distribution measurement: cigarette smoke, fine dust, and pollen.^10^ The purpose of the study presented here was to develop and apply an alternative method of evaluating a recirculating air purification unit, the Molekule Air Mini, in terms of both its size‐dependent particle collection efficiency as well as in its efficacy in removing and inactivating virus‐laden aerosols with three different viruses: an influenza A virus, an alphacoronavirus, and a betacoronavirus. The tested recirculating air purification unit utilizes photoelectrochemical oxidation in addition to typical mechanical filtration in pollutant removal. It was thus important to evaluate collection of virus‐laden particles as well as to evaluate the inactivation of virus‐laden particles passing through the unit. The wind‐tunnel method, which has not been applied previously in evaluating recirculating air purifiers, involves mounting and sealing the air purification unit into a single pass wind tunnel, and examining both size‐dependent particle collection efficiency as well as virus removal efficiency in single pass measurements. In this regard, the developed approach heavily hinges upon requirements of the ASHRAE 52.2 method of evaluating filters for HVAC ducts. For viruses, we distinguish between collection and inactivation via a combination of upstream and downstream sample titer and reverse transcription‐quantitative polymerase chain reaction (RT‐qPCR) measurements. Physical collection efficiency is quantified as a function of size as the penetration (1‐collection efficiency), while virus removal is quantified via log reduction (based 10 log of the upstream‐to‐downstream concentration ratio). The most common figure of merit for recirculating air purification technologies is the clean air delivery rate (CADR), which is the product of the devices collection or removal efficiency and its operating volumetric flow rate. Wind tunnel testing at prescribed flow rates enables determination of the CADR in a size‐dependent manner. The methods employed and results with the tested air purification unit are presented in the following sections. Subsequently, we discuss the implications of measurement results, including future prospects in studying virus aerosol control technologies.

## 2 MATERIALS AND METHODS

Three types of experiments were performed to examine the air purification unit's capabilities in removing viable viruses from an aerosol. First, standard particle penetration tests with a non‐viable test aerosol (KCl particles) were carried out. Second, virus penetration tests were carried out using bovine coronavirus (BCoV, a betacoronavirus), porcine respiratory coronavirus (PRCV, an alphacoronavirus), and a swine influenza A virus (IAV) of human origin. All tested viruses are pleomorphic; for the coronaviruses, size estimates range from 65 nm −210 nm,^11^ while for IAV size estimates fall in the 80 nm–130 nm range.^12^ Through the aerosolization approach employed, viruses are incorporated into larger particles which are composed of residue salts and small molecules from the nebulized virus stock suspensions.^13^ To our knowledge, only with electrospray‐based aerosolization have isolated viruses been measured in an aerosol.^14^, ^15^ The particular coronaviruses and the influenza virus used were selected because of recent concern of aerosol routes to infection^3^, ^16^, ^17^ by viruses in these families (coronaviridae and orthomyxoviridae), but at the same time, their uses do not require Biosafety Level III facilities for experiments. In examining two different types of coronaviruses, we specifically investigate whether different coronavirus genera display different inactivation behavior. Virus titration, RT‐qPCR, and fluorimetry (of a fluorescent tracer) were utilized to characterize virus removal. Third, virus titration and RT‐qPCR assays were performed on BCoV directly loaded onto the photoelectrochemical filter utilized in the test air purification unit, along with selected control filters. We briefly describe the methods employed in each of these measurements.

### 2.1 Wind tunnel penetration tests

**FIGURE 1:**
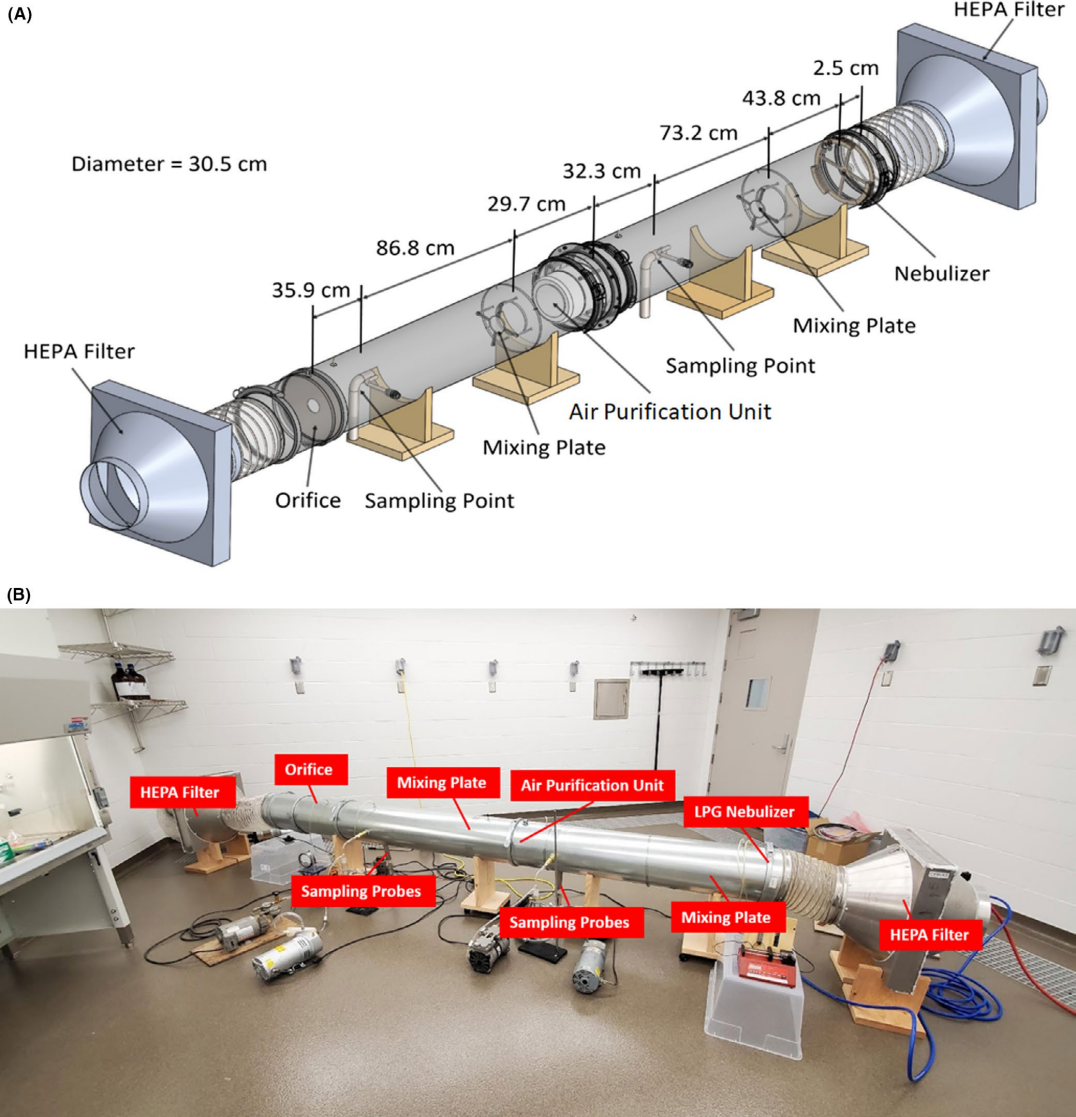
A schematic diagram of the wind tunnel system utilized in penetration and log reduction measurements with the air purifier unit installed (A). Labeled photograph of the wind tunnel system within a biosafety level II room (B)

Both non‐infectious particle and viable virus penetration tests were performed using a custom‐designed wind tunnel, depicted in Figure 1, and described in detail in Qiao et al (2020).^18^ The air purification unit (Molekule Air Mini, Molekule Inc.,) was inserted into the test wind tunnel and sealed with a silicone adhesive to a mounting plate designed specifically for the air purification unit. The tested unit control technology incorporates a minimum efficiency reporting value‐16 (MERV‐16)‐based filter laminate including a photoelectrochemical oxidation (PECO) layer, which is exposed to UV‐A light during operation to drive oxidation of incoming particles and vapor phase species. The filter laminate is structured such that the outer surface of the filter (the upstream layer) features a carbon and electrostatic media, providing the bulk of the physical particle filtration. The inner surface of the filter (the downstream layer) features the media upon which the PECO reaction takes place. The UV‐A source is only turned on when the unit is closed with a manufacturer‐produced filter installed, and the tested unit used in this study was not modified in any way. Sealing around the unit effectively drove all flow in the wind tunnel through the air purification unit (entering its inlet and exiting its outlet, as would be the case in recirculating operation), enabling penetration tests akin to ASHRAE Standard 52.2 tests for duct filtration units. For non‐infectious particle penetration tests, we operated the wind tunnel at flow rates of 566 L/min^−1^, 1416 L/min^−1^, and 2350 L/min^−1^. We utilized a custom pneumatic nebulizer^19^, ^20^ to nebulize 5% weight aqueous KCl solution, yielding polydisperse KCl particles in the 10 nm to 10 µm diameter range. The produced droplets were entrained into the wind tunnel flow; droplet drying yielded dry KCl particles at a relative humidity in the 57%–62% range near 300 K. The size distribution function of KCl particles was measured upstream of the tested unit by sampling with isokinetic probes into both a TSI 3034 scanning mobility particle sizer (SMPS), which integrates a differential mobility analyzer and condensation particle counter for particle size distribution measurements in the 10 nm–487 nm range, and a TSI 3330 optical particle spectrometer (OPS), optically measuring particle size distributions in the 500 nm–10 µm range (we note down to 300 nm is possible, but we applied the OPS only at sizes beyond the SMPS range). A separate Po‐210 bipolar ion source was used upstream of the SMPS to bring particles to a steady‐state charge distribution prior to measurement. Similar size distribution measurements were made downstream of the air purification unit with both the SMPS and the OPS where five upstream measurements and four downstream measurements were made with the OPS, and four upstream measurements and five downstream measurements with the SMPS, with upstream and downstream measurements taken in an alternating fashion. Four particle penetration values were then calculated following the ASHRAE Standard 52.2 guidelines for alternating upstream and downstream concentration measurements in the 10 nm–10 µm size range, at the three test flow rates. Example upstream size distribution functions and further details on penetration calculations are provided in the Figure S1 and its caption. All tested flow rates were accessible directly with the air purifier unit operating as a stand‐alone instrument with 2350 L/min^−1^ achieved at its highest speed setting (there are five settings); however, because of the additional pressure drop brought about by wind tunnel components, an external blower was used to boost the system flow rate to the desired value for each test, with the flow rate monitored using a calibrated orifice meter. For both the upstream and the downstream sampling, customized mixing plates were installed before the sampling points to redistribute the particles across the radius of the wind tunnel. Prior to testing, via the Log‐Tchebycheff method for both flow velocity and particle concentration, the coefficient of variation in the flow velocity was found to be below 0.10 at the location of the first upstream sampling point, and for particles below 5 µm in diameter, the coefficient of variation in particle concentration was below 0.15. Background correction was not applied in penetration calculation because background particle levels were multiple orders of magnitude below concentrations when aerosolizing KCl particles. Correlation testing of particle concentration in the absence of the air purification unit from the upstream to downstream sampling locations yielded a statistically insignificant difference from 1.0; hence, a correlation correction was also not applied to reported penetration values.

The wind tunnel system was operated similarly for virus penetration tests, but with the entire tunnel housed within a biosafety level II facility in the Veterinary Isolation Building at the University of Minnesota Saint Paul Campus (as depicted in Figure 1B) and following the procedures approved by the University of Minnesota Institutional Biosafety Committee protocol number 1808‐36316H. Air was entrained into the wind tunnel by an external blower connected downstream, passing through HEPA filters (Air Filters, Inc. Model MPH24242MCUG2) both at the inlet and prior to the blower. The flow rate was monitored via continuous measurement of the pressure drop across a calibrated thin plate orifice meter with an opening diameter of 6.27 cm, positioned prior to the downstream HEPA filter. Sampling probes with diameters of 3.47 cm were placed at both sampling locations at the center of the duct and aligned facing the flow direction with sampling carried out via Andersen cascade impactors.^21^ Stages 0, 5, and 6 and after filter were installed in both impactors with vacuum pumps facilitating sampling at 90 L/min^−1^. At this flow rate, the sampling probes were not isokinetic (they operated at higher velocities than the wind tunnel). However, based on the aforementioned wind tunnel correlation and uniformity tests, and the similarity of the upstream and downstream sampling systems, we do not believe non‐isokinetic sampling influenced the results in this study. While alternative samplers have been proposed and tested in prior studies examining virus aerosol collection,^13^, ^22^, ^23^, ^24^, ^25^, ^26^ at present, in evaluating the efficacy of control technologies for viruses in aerosols, we find the critical issue remains maximizing sampler flow rate and collection efficiency^26^, ^27^, ^28^ in the appropriate size range for virus‐laden particles, hence smaller cut‐point stages in Andersen impactors^29^ operated at non‐traditional elevated flow rates were selected for this study. Evident in Figure 1, SKC BioSamplers (SKC Inc., Eighty Four) were also installed and operated for sampling at 10 L/min^−1^ both at the upstream and downstream sampling locations. However, as the solution extracted from these samplers yielded appreciably lower titers, RNA concentrations, and fluorescein concentrations both upstream and downstream for all tests, BioSampler results were not used in evaluating air purifier efficacy and only to confirm virus and RNA presence or absence. To commence virus aerosolization challenges, the wind tunnel was first turned on and checked on a daily basis to make sure it operated under negative pressure with respect to the ambient, such that air would be pulled into it in case of accidental leaks, and filtered by the HEPA filter without having viruses penetrate into the surrounding room. The air purifier unit was turned on next, set to speed setting 3 (of 5), and the air flow rate of the wind tunnel was adjusted such that the flow rate through the air purification unit was monitored at 1516 L/min^−1^ for all tests. With simultaneous upstream and downstream sampling, upstream of the wind tunnel the flow rate was 1616 L/min^−1^, with 100 L/min^−1^ diverted to samplers (the Andersen Impactor and BioSampler), and another 100 L/min^−1^ downstream diverted to samplers; hence, the orifice meter monitored 1416 L/min^−1^ during wind tunnel operation. A compressed air pump (Porter Cable, Jackson, TN) was then turned on, regulated at 138 kPa for operation of the pneumatic nebulizer (identical to that used in non‐infectious penetration tests) containing high titer virus suspensions. Dispersion air for the nebulizer was controlled and monitored at 1.5 L/min^−1^. A bipolar corona discharge source was used to generate roughly equal concentrations of positive and negative ions in the dispersion air. In separate tests, we found that the corona discharge produced a negligible amount of ozone, and its use did not statistically significantly influence virus viability. Rather, bipolar ions were utilized to bring produced particles to a near steady‐state bipolar charge distribution,^30^ minimizing the potential for electrostatic particle losses. Prior studies have additionally shown viable viruses downstream of a variety of ionization schemes (including after electrospray ionization)^14^, ^15^, ^31^, ^32^; hence, combining results here and in prior work, it does not appear ionization of virus‐laden particles has a demonstrated influence on virus viability in aerosols. In total, the applied aerosolization procedure yielded bipolarly charged, virus‐laden highly polydisperse particles which were near 3 µm in mean diameter by volume; hence, the virus‐laden particles were largely supermicrometer in diameter but primarily below 5 µm, as shown in Qiao et al.^18^

For virus aerosolization suspensions, the three test viruses were prepared as described in the Supplementary material. Viruses were grown to titers of 10^7^–10^7^.^75^ TCID_50_ ml^−1^ (50% tissue culture infectious dose) for BCoV, PRCV, and IAV. All virus suspensions were also spiked with 0.3 g/L^−1^ fluorescein dye, used as a physical tracer for particle penetration. Upstream and downstream sampling were carried out for 30 min in triplicate for each test virus, both in the presence (penetration tests) and absence (correlation tests) of the air purification unit in the wind tunnel. For correlation tests, flow was controlled entirely by the external blower in the wind tunnel, with flow rate held at the same value as in penetration tests. Following each 30‐minute aerosolization test, the blower was turned off, the syringe pump stopped, and the particles which deposited on the impactor plates were extracted with a cell scraper using 3 ml of appropriate virus growth media on each stage as collection liquid. All three stages were pooled for a single titer and RT‐qPCR measurement per impactor (though with the filters kept separate from the impaction stages). This was done in an effort to maximize signal in all measurements, and avoid any issues in data interpretation brought about by particle bounce at the elevated impactor flow rates. Virus titration, RT‐qPCR, and fluorimetry of impaction plate samples and total filter samples were carried out as described in the “*Virus Titration*,*RT*‐*qPCR*, *and* *Fluorimetry”* subsection.

### 2.2 Virus viability on filter media

In addition to carrying out measurements on aerosol samples, in tests with the air purification unit, samples were collected from the upstream and downstream filter laminate layers by wiping the filter with a 3 inch × 3 inch gauze piece wetted with virus growth media.^33^ During the air purification unit operation, the inside of the filter laminate is exposed to a UV‐A light source; UV‐A photons and the PECO layer material drive photoelectrochemical oxidation of deposited particulate matter and material passing through the device.^34^ Subsequent to swab tests, we elected to perform a systematic study of the influence of both the filter material and UV‐A irradiation on the viability of viruses on filter surfaces. 0.5 ml of a high titer (10^7.25^ TCID_50_ ml^−1^) suspension of BCoV was inoculated onto 1 inch × 2 inch cut pieces of (1) the PECO filter conventionally used in the air purification unit (2 sets, 1a and 1b), (2) the filter conventionally used in the air purification unit, but without the PECO layer, and (3) commercial HEPA filtration media. We remark that the Molekule Air Mini will not operate without manufacturer‐designated filters installed; hence, type (1) and type (2) filters were provided by the manufacturer, with type (2) made specifically for these experiments. Type (1a) filter pieces were re‐inserted into the filter laminate, placed within the air purifier unit and the device operated for 4 h. At time intervals of 0, 30, 60, 120, and 240 min, three of the filter samples were extracted. Following extraction, the removed filter samples were replaced by new filter strips in an effort to minimize any changes to the flow pattern in the air purification unit. Type (1b) filters were similarly extracted at these time intervals, but were not placed within the air purification unit and hence were not exposed to UV‐A light. Type (2) filter samples were placed within the device and followed the procedure for type (1a) samples; however, photoelectrochemical oxidation is not anticipated in this instance as the PECO material is excluded in type (2). Type (3) HEPA filter samples were handled in an identical manner to type (1b) samples. Extraction was carried out by placing each filter sample into a 50 ml conical tube with 5 ml BCoV growth media, vortexing for 2 min at 3,000 rpm, and centrifuging at 3000 × *g* for 10 min at 4°C. The supernatant containing the virus was then aliquoted into tubes. Samples were titrated on the same day as collection, and the remaining aliquoted samples were frozen at −80°C until RT‐qPCR and fluorimetry tests.

### 2.3 Virus titration, RT‐qPCR, and fluorimetry

Pooled Andersen impactor stages and final stage filter samples were used to measure upstream and downstream concentrations of (1) viable viruses, carried out by virus titration, (2) nucleic acids, carried out by RT‐qPCR, and (3) fluorescein, carried out by fluorimetry. However, as Andersen impactor final stage filter samples did not yield detectable viable viral concentrations, we did not utilize these results in discussing the air purification unit performance. Viable viruses from pooled Andersen impactor stages were titrated in specific cells (see Supplementary material) on the same day as collection, using the 50% tissue culture infectious dose (TCID_50_) method. Samples were ten‐fold serially diluted from 10^−1^ to 10^−7^ in the appropriate virus growth media; then, aliquots of 100 µl of each sample dilution were plated into four replicate wells of 24‐h‐old cell monolayers in 96‐wells cell culture plates. Plates were incubated at 37°C with 5% CO_2_. Virus titer results were calculated using the Karber method^35^ by observing the cytopathic effects under inverted microscope after 7 days of incubation for BCoV, and 5 days of incubation for PRCV and IAV. Virus titer results were expressed as TCID_50_ ml^−1^. Fifty microliters of sample were used for viral RNA extraction with the MagMAX^TM^ −96 Viral RNA Isolation kit (Applied Biosystems by Thermo Fisher Scientific, Lithuania) according to the manufacturer's instructions, on a semi‐automatic MagMAX Express‐96 Deepwell Magnetic Particle Processor (Applied Biosystems by Thermo Fisher Scientific). The RNA was eluted with 50 μl of elution buffer and stored at −80°C until used for viral genome quantification.

For BCoV RT‐qPCR, we used primers and probe targeting the transmembrane gene as described in Decaro et al., 2008.^36^ The Ambion AgPath‐ID One‐Step RT‐PCR kit (Applied Biosystems by ThermoFisher) was used for the BCoV RT‐qPCR reaction mixture (25 µl), consisting of 5 μl of template RNA, 12.5 μl of 2X RT‐PCR buffer, 1 μl 25X RT‐PCR Enzyme Mix, 0.5 μl 10 μM forward primer (200 nM final concentration), 0.5 μl 10 μM reverse primer (200 nM final concentration), 0.3 μl 10 μM probe (120 nM final concentration), and 5.2 μl nuclease‐free water. BCoV RT‐qPCR was performed in the ABI7500 Fast Real‐Time PCR thermocycler system (Applied Biosystems, Foster City) with the following thermocycling conditions: 45°C for 10 min, 95°C for 15 min followed by 45 cycles of 95°C for 15 s, and 58°C for 45 s. In each run of RT‐qPCR, ten‐fold serial dilutions of standard curve samples with known concentration and no template control were used as positive and negative controls, respectively. The absolute viral genome copy numbers, expressed as genome copies ml^−1^, were calculated using the cycle threshold (Ct) values and the standard curves. For the PRCV RT‐qPCR, we used an in‐house developed PCR primers and probe set and RT‐PCR conditions as described in Qiao et al., 2020.^18^ We used primers and probe targeting the IAV matrix gene as described in Slomka et al., 2010,^37^ and IAV RT‐qPCR conditions described in Nirmala et al., 2021.^38^

Samples of pooled Andersen impactor plates were used for the fluorimetry test. One hundred microliters of sample were aliquoted into 96‐wells black plates (Thermo Fisher Scientific), sealed with a transparent film to prevent evaporation, and results were read in a micro‐plate reader (Model Synergy H1, BioTek). Excitation and emission wavelengths of 485 nm and 515 nm respectively, with a gain of 30, were used to measure the fluorescence intensity.

## 3 RESULTS & DISCUSSION

### 3.1 Non‐infectious particle penetration tests

**FIGURE 2:**
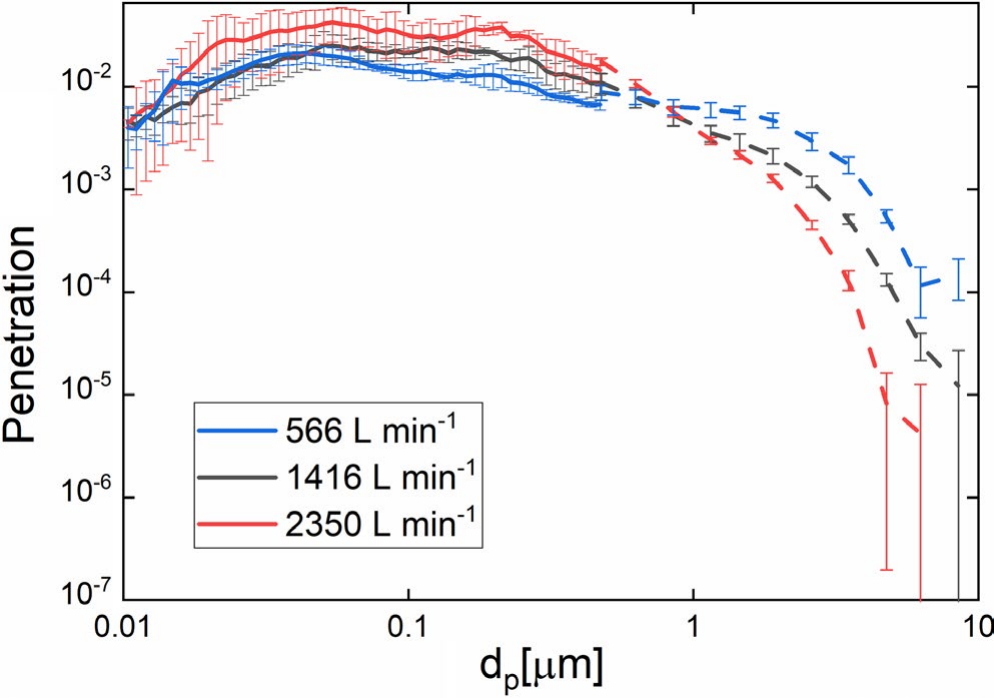
Particle penetration versus particle diameter for the air purification unit, measured in a sealed wind tunnel. Solid lines denoted measurements utilizing a scanning mobility particle sizer, while dashed lines denote optical particle sizer results. The error bars are the standard deviation according to the four penetration values at each particle size determined following ASHRAE Standard 52.2 guidelines

Particle penetration, that is, the ratio of the downstream size distribution function to upstream size distribution function (1‐ collection efficiency), is shown in Figure 2 in the 10 nm to 10 µm diameter range for three selected flow rates. Solid lines denote data obtained with an SMPS, while dashed lines denote OPS measurement results. While the two instruments focus on different size ranges (with 0.5 µm to 10 µm plotted for the OPS), evident for data at all three test flow rates is that the penetration results from both instruments combine to form near‐singular curves across the entire measured size range, with little discontinuity at the SMPS‐OPS interface near 0.5 µm. As expected for a MERV 16‐based filter,^39^ the most penetrating size falls below 300 nm and is in the 40–60 nm size range. Even in this size range, the penetration is below 10^−1^ and the tested air purifier penetration is below 5 × 10^−2^ (> 95% efficient) at nearly all sizes examined. Above 0.5 µm, with the exception of the highest flow rate setting tested, the penetration is below 10^−2^. The observed variation in penetration curves with flow rate is consistent with traditional fibrous filtration efficiency. For supermicrometer particles, collection is facilitated by particle inertial impaction and interception, leading to lower penetrations at increasing flow rate.^40^, ^41^ When inertial impaction becomes a significant mechanism, as is the case for higher flow rates and larger particles, the penetration decreases to 10^−4^–10^−5^. Meanwhile, submicrometer particles are predominantly collected via diffusion,^42^, ^43^ leading to higher penetrations with increasing flow rate in the submicrometer size range. The size‐dependency of these disparate collection mechanisms leads to the most penetrating particle size falling into the 30–200 nm size range.^44^

While low penetration values along these lines are expected for MERV 16 filters, we believe it is important to measure such collection efficiencies directly for recirculating air purification units, and propose doing so in the manner described in this work is the most accurate method of collection efficiency measurement. While extremely efficient filters and other control technologies are commonly incorporated into recirculating air purifiers, Waring et al (2008)^45^ show that recirculating air purifiers incorporating higher efficiency HEPA filters and electrostatic precipitators may still have effectively high penetrations (i.e. >0.50, and consequently collection efficiencies below 0.50) because of poor sealing (leaks) within the units (with leaks frequently termed “bypass” flow). Furthermore, Waring et al (2008)^45^ infer collection efficiency from particle concentration decay tests wherein they first calculate a size‐dependent CADR for the tested air purifiers and infer the collection efficiency from the ratio of CADR to operating flow rate. While qualitatively, this method can identify instances where bypass flow is present and devices have penetrations higher than anticipated for the control technologies incorporated, collection efficiency curves inferred in this manner (i.e. room tests with separate aerosol “loading” and “collection” intervals) typically have unphysical oscillations and can lead to unphysical values in excess of 100% for collection efficiency. The results of particle concentration decay tests are also inevitably dependent upon the dimensions of the room utilized, deposition within the room,^46^ and the ability to create spatially uniform particle concentration profiles over the course of particle concentration decay measurements. Direct collection efficiency measurement is a much faster test enabling appropriate counting statistics across a wide size range, proper numbers of replicates, and the CADR is then simply calculated by the product of the measurable operating flow rate and collection efficiency. In the case of the air purification unit tested here, because of the low penetration values obtained, the bypass flow is negligible, and the CADR is approximately equivalent to the operating flow rate for particles which potentially contain infectious viruses.

### 3.2 Virus aerosol removal

Viruses in aerosols, which are attached to sub‐ and supermicrometer aerosol particles,^3^, ^17^, ^23^, ^47^ will be removed by recirculating air purification units in a similar fashion to non‐infectious particles, that is, biological activity has little‐to‐no influence on the physics governing particle penetration. Nonetheless, it is important to examine virus aerosol removal by air purification units for several reasons. First, in addition to collection, technologies incorporating catalytic systems or which generate reactive oxygen species may not only collect virus‐laden particles, but may facilitate the inactivation of virus‐laden particles passing through them. Collection and inactivation can be distinguished from one another via the simultaneous use of virus titration to detect upstream and downstream viable viruses, RT‐qPCR to detect viral RNA, and assays for an independent physical tracer (eg fluorescent dye) to examine physical removal on a mass basis. Differences between virus titer‐based measurements and either the RT‐qPCR or physical tracer measurements provide evidence for the inactivation of uncollected viruses by the tested unit; if the titer‐based removal is largest, then the difference in log reduction on a titer‐basis and physical tracer basis is the inactivation log reduction.^31^ Furthermore, differences between RT‐qPCR and physical tracer results provide evidence for RNA degradation in particles passing through the unit, which should also coincide with inactivation. Such information cannot be obtained by physical collection efficiency measurements alone. Second, the collection of virus‐laden particles will presumably yield viable viruses on the collection surface. The viability and change in viability over time of collected viruses are also of interest, as filters need to be cleaned or exchanged. Third, in carrying out virus aerosol tests, through control tests (ie measurements without the test unit installed), the influence of aerosolization and transport in an aerosol (including droplet drying) on virus viability is assessed; little remains known about how the act of aerosolization itself influences virus viability, and the extent to which aerosol‐based transmission contributes significant to infection by different virus species is an ongoing topic of investigation.^1^, ^2^, ^3^, ^49^

**FIGURE 3:**
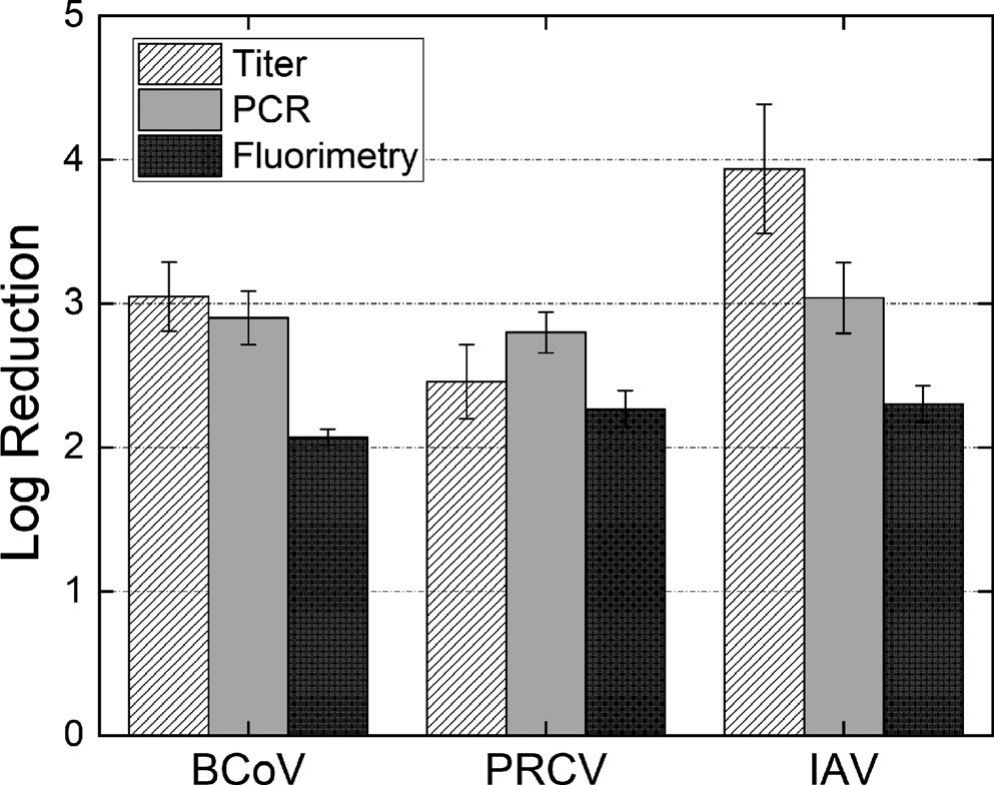
Log reductions in virus concentrations (log_10_[upstream concentration/downstream concentration] with concentration based upon fluorescein tracer (physical removal), RT‐qPCR (RNA physical removal and damage), and virus titration (viable viruses) for bovine coronavirus (BCoV), porcine respiratory coronavirus (PRCV), influenza A virus (IAV). The error bars are the standard deviation of the log reduction calculated by root sum square error propagation method applying the standard deviations of the upstream and downstream triplicate measurements

For the three test viruses examined, consistent with prior characterization efforts for bioaerosol control technologies, results are reported in terms of log reduction, defined as the base 10 logarithm of the ratio of the upstream sampled concentration to downstream sampled concentration.^31^ Log reductions are reported for measurements based upon virus titer, RT‐qPCR, and fluorimetry of pooled‐impactor stage samples. Error bars represent the standard deviation based upon the root sum square method and triplicate upstream and downstream measurements. Table 1 additionally summarizes all directly measured fluorimetry results, virus titers, and RT‐qPCR concentrations measured for both tests including the tested air purification unit (penetration tests) as well as triplicate tests in the absence of the air purification unit (control tests, to ensure minimal sample loss from the upstream to downstream sampler). No systematic change in concentrations quantified by any of the three measurement types was discernable in control tests; hence, the Figure 3 log reductions are not corrected by control test penetrations.

**TABLE 1:** A summary of fluorimetry, virus titer, and RT‐qPCR test results for pooled Anderson impactor stages upstream and downstream of the air purification unit in the wind tunnel

Replicate	BCoV		PRCV		IAV	
	Upstream	Downstream	Upstream	Downstream	Upstream	Downstream
Fluorescein Correlation Control Tests [a.u.]						
1	9.20 × 10^2^	1.07 × 10^3^	6.96 × 10^2^	9.29 × 10^2^	5.24 × 10^2^	1.13 × 10^3^
2	1.00 × 10^3^	1.08 × 10^3^	7.45 × 10^2^	3.70 × 10^2^	1.12 × 10^3^	1.15 × 10^3^
3	1.06 × 10^3^	1.08 × 10^3^	7.27 × 10^2^	7.51 × 10^2^	1.07 × 10^3^	1.17 × 10^3^
Average	9.95 × 10^2^	1.08 × 10^3^	7.23 × 10^2^	6.83 × 10^2^	9.03 × 10^2^	1.15 × 10^3^
Fluorescein Penetration Tests [a.u.]						
1	1.05 × 10^3^	9.00	7.31 × 10^2^	4.00	9.33 × 10^2^	4.00
2	1.24 × 10^3^	10.00	9.97 × 10^2^	6.00	7.53 × 10^2^	3.00
3	1.01 × 10^3^	9.00	8.62 × 10^2^	4.00	7.22 × 10^2^	5.00
Average	1.10 × 10^3^	9.33	8.63 × 10^2^	4.67	8.03 × 10^2^	4.00
Virus Titer Correlation Control Tests [TCID50 ml^−1^]^a^						
1	1.78 × 10^6^	3.16 × 10^6^	1.78 × 10^3^	5.62 × 10^3^	3.16 × 10^6^	5.62 × 10^6^
2	1.48 × 10^6^	1.78 × 10^6^	3.16 × 10^3^	5.62 × 10^3^	3.16 × 10^6^	3.16 × 10^6^
3	3.16 × 10^6^	3.16 × 10^6^	1.00 × 10^3^	5.62 × 10^3^	1.78 × 10^6^	1.00 × 10^6^
Average	2.14 × 10^6^	2.70 × 10^6^	1.98 × 10^3^	5.62 × 10^3^	2.70 × 10^6^	3.26 × 10^6^
Virus Titer Penetration Tests [TCID50 ml^−1^]^a^						
1	1.00 × 10^6^	5.62 × 10^2^	1.78 × 10^4^	3.16 × 10^1^	1.78 × 10^6^	1.78 × 10^2^
2	3.16 × 10^5^	5.62 × 10^2^	1.78 × 10^4^	5.62 × 10^1^	1.00 × 10^6^	5.62 × 10^1^
3	5.62 × 10^5^	5.62 × 10^2^	5.62 × 10^3^	5.62 × 10^1^	1.00 × 10^5^	1.00 × 10^2^
Average	6.26 × 10^5^	5.62 × 10^2^	1.37 × 10^4^	4.80 × 10^1^	9.59 × 10^5^	1.11 × 10^2^
RT‐qPCR Correlation Control Tests [Copies ml^−1^]						
1	3.60 × 10^8^	4.26 × 10^8^	1.58 × 10^7^	2.13 × 10^7^	2.88 × 10^9^	2.62 × 10^9^
2	4.39 × 10^8^	4.82 × 10^8^	1.40 × 10^7^	1.73 × 10^7^	2.66 × 10^9^	2.39 × 10^9^
3	4.36 × 10^8^	4.70 × 10^8^	1.36 × 10^7^	1.58 × 10^7^	2.50 × 10^9^	3.08 × 10^9^
Average	4.12 × 10^8^	4.59 × 10^8^	1.45 × 10^7^	1.81 × 10^7^	2.68 × 10^9^	2.70 × 10^9^
RT‐qPCR Penetration Tests [Copies ml^−1^]						
1	3.51 × 10^8^	3.50 × 10^5^	3.53 × 10^7^	4.99 × 10^4^	1.64 × 10^9^	1.62 × 10^6^
2	4.90 × 10^8^	6.10 × 10^5^	5.35 × 10^7^	7.87 × 10^4^	8.23 × 10^8^	5.79 × 10^5^
3	2.60 × 10^8^	4.23 × 10^5^	3.80 × 10^7^	7.31 × 10^4^	1.33 × 10^9^	1.26 × 10^6^
Average	3.67 × 10^8^	4.61 × 10^5^	4.22 × 10^7^	6.72 × 10^4^	1.27 × 10^9^	1.15 × 10^6^

Note

Measurements were carried out in triplicate, both without the air purification unit installed (control/correlation tests) and with the air purification unit installed (penetration tests).

Abbreviations: BCoV, bovine coronavirus; IAV, influenza A virus; PRCV, porcine respiratory coronavirus.

^a^ Values of 3.16 × 10^1^ correspond to virus titer levels below the limit of detection, with this value utilized as the upper limit of the possible virus titer.

Fluorimetry results for all three test viruses yield log reductions in excess of 2.0; this is anticipated as the mass‐median diameter of particles using all virus suspensions is in the supermicrometer size range, where the penetration is between 10^−2^ and 10^−3^. Meanwhile, RT‐qPCR results yield log reductions in excess of 2.5 for both tested coronaviruses, and above 3.0 for IAV. The log reduction results based upon virus titers for BCoV and PRCV are similar to the RT‐qPCR log reduction results, suggesting that if uncollected viruses are additionally inactivated by photoelectrochemical oxidation within the tested air purification unit, inactivation additionally drives damage of the RNA fragment selected for amplification in the RT‐qPCR assay. More pronounced differences between log reduction results for fluorimetry, RT‐qPCR, and titer are observed for IAV; there is nearly a 0.75 log reduction increase from fluorimetry to RT‐qPCR, and then nearly a 1.0 log reduction increase from RT‐qPCR to virus titer. Therefore, measurement results for IAV suggest that not only are virus‐laden particles collected in the air purification unit but also virus within uncollected particles are efficiently inactivated during passage through the air purification unit. Although determination of the mechanism of inactivation for viruses is beyond the scope of this study, the unique results for IAV relative to the tested coronaviruses suggest that studies employing surrogate viruses toward collection and inactivation measurements should select surrogate viruses judiciously, as inactivation results can clearly be virus family‐dependent. This stated the similarity in performance of the two tested coronaviruses, from different genera, strongly suggests that similar results would be obtained for SARS‐CoV‐2 and others in the coronavirus family. We also note that because of the inherent variability in virus titer measurements, and to a lesser extent RT‐qPCR measurements, small differences in log reduction (e.g. 0.1–0.2) are not strong evidence of inactivation.

### 3.3 Virus viability on filter media

**FIGURE 4:**
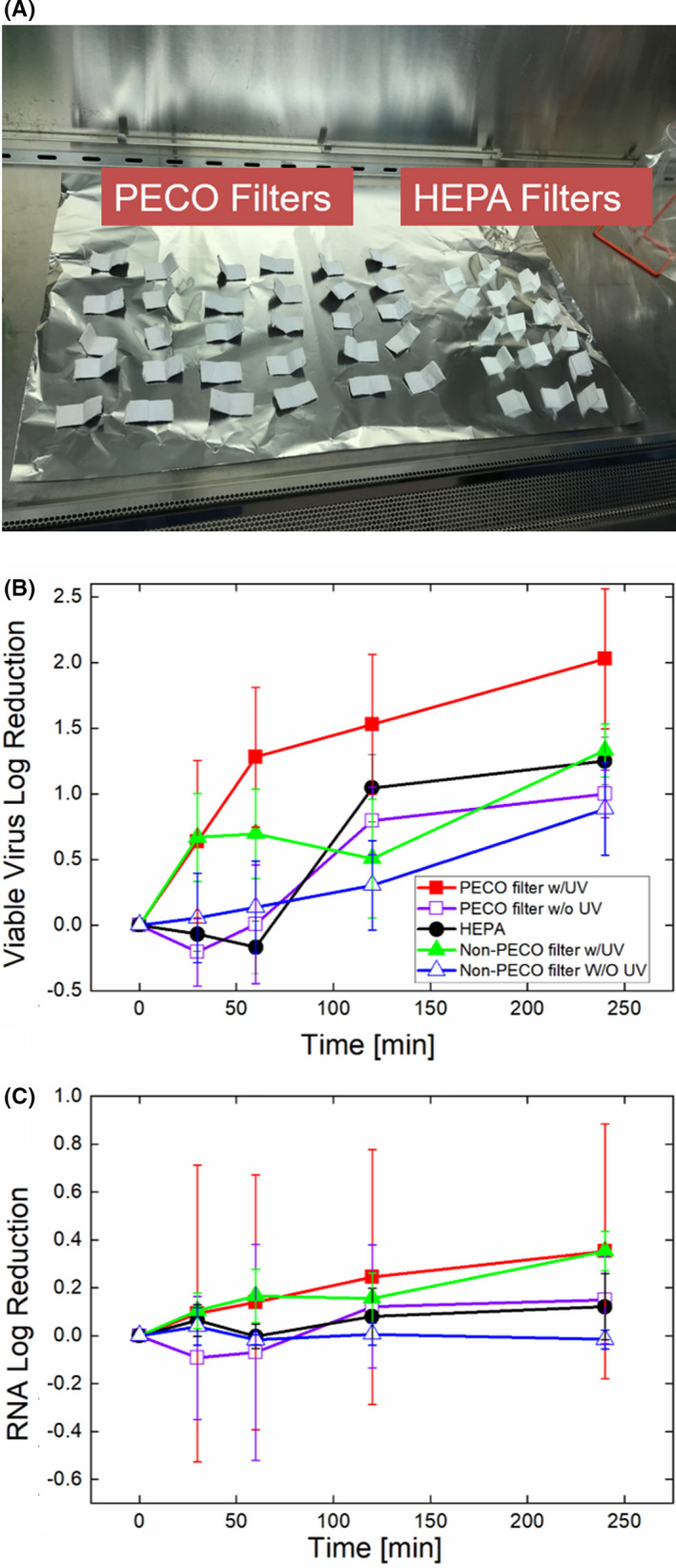
Images of 1 in × 2 in cutout strips of photoelectrochemical oxidation (PECO) filter laminates and HEPA filter media used in virus surface inactivation studies (A). Log reduction over time on various filter media based upon bovine coronavirus titer (B). Log reduction over time on various filter media based upon bovine coronavirus RT‐qPCR (C). Log reduction was calculated based on the average values of three replicates at different sampling times, comparing with initial time (t = 0) in both titer and RT‐qPCR measurements. Error bars were calculated using the root sum square error propagation method with the standard deviations for triplicate measurements at the initial time and the sampling time in titer and RT‐qPCR considered, respectively

Swab extractions from the outside and inside of the PECO filters following virus aerosol penetration tests yielded the virus titers and RT‐qPCR RNA concentrations displayed in Table 2. Each displayed value is the result after triplicate measurements for each virus, that is after the filter had been exposed to aerosols for 90 min produced from high titer suspension nebulization. Viral RNA was detected on both the outside and the inside of filters under all conditions, though with appreciably less on the inside. This is part due to asymmetric loading along the filter depth which is common place in fibrous filtration; however, interestingly, for all conditions, the inside of the filter did not yield any viable virus during titration, as the titer amount noted correspond to titers below the limit of detection (for which a TCID_50_ ml^−1^ of 3.16 × 10^1^ is assigned, by default). Because of this finding, we elected to examine the influence of both the PECO filter material and potential PECO reaction on virus viability using filter swatches which feature all filter layers instead of only simply sampling the upstream and downstream filter surfaces after single pass efficiency tests. The log reduction in BCoV viability over time and log reduction in detected RNA concentration over time are plotted in Figure 4, which also displays images of PECO and HEPA filter samples. Evident in Figure 4B, the PECO filter material alone, the filter without the PECO layer both inside and outside the air purification unit, and the HEPA filter all yielded similar changes in virus titer over time; a log reduction near 1.0 is achieved after 240 min on the filter. Meanwhile, for the PECO filter exposed to UV‐A light within the air purification unit, a log reduction in virus viability near 2.0 is observed over the same time interval, with discernable increases in the log reduction evident for test periods longer than 60 min. This provides evidence that for collected viruses, photoelectrochemical oxidation can drive virus inactivation at a faster rate, presumably through the product of short‐lived reactive oxygen species (which also drive oxidation of volatile organic compounds (VOCs)) passing through the PECO filter system.^34^ Shown in Figure 4C, reduction in BCoV viability is not associated with decay in the RNA fragment used in RT‐qPCR amplification, which is similar for all tested virus types. This is presumably because the RNA fragment chosen for amplification is rather short (number of bases), and the oxidation reactions driving inactivation act non‐specifically.

**TABLE 2:** Summary of RT‐qPCR, virus titer results of bovine coronavirus (BCoV), porcine respiratory coronavirus (PRCV), and influenza A virus (IAV) for extraction from inside and outside of the photoelectrochemical oxidation (PECO) filters during operation of the air purification unit

Virus	RT‐qPCR [Copies ml^−1^]		Titer [TCID50 ml^−1^]	
	Inside	Outside	Inside	Outside
BCoV	9.38 × 10^4^	8.49 × 10^8^	3.16 × 10^1^ ^a^	1.78 × 10^6^
PRCV	9.25 × 10^3^	2.15 × 10^8^	3.16 × 10^1^ ^a^	5.62 × 10^4^
IAV	3.47 × 10^4^	5.31 × 10^9^	3.16 × 10^1^ ^a^	5.62 × 10^5^

Note

The filters were loaded for 90 min prior to sampling for each virus.

^a^ Values of 3.16 × 10^1^ correspond to virus titer levels below the limit of detection, with this value utilized as the upper limit of the possible virus titer.

## 4 CONCLUSIONS and LIMITATIONS

We have developed and applied a medium‐scale wind tunnel toward examining the size‐dependent physical collection efficiency and virus removal efficiency (accounting for removal and inactivation) of a recirculating air purification unit, the Molekule Air Mini. Wind tunnel testing was performed using IAV, BCoV, and PRCV. Based on the performed measurements, we make the following concluding remarks and note the following limitations of our study:

1. Wind tunnel systems enable direct measurement of the single‐pass collection efficiency for recirculating air purification systems. While wind tunnel systems would require modification to test different air purification units because of the need to seal devices in a manner enabling flow into the inlet upstream and out the outlet downstream, we argue this method of testing devices will be more reliable than more commonly employed clean air delivery rate tests,^45^, ^50^ which involve particle concentration decay measurements in a room. Properly designed wind tunnels, with uniform velocity and particle concentration profiles, should yield results with minimal wind tunnel‐to‐wind tunnel variability in testing. Wind tunnel usage also obviates the need to develop enclosed rooms and aerosolization procedures approximating “well‐mixed” conditions, as well as the need to correct results for the influence of particle clearance by ventilation, gravitational settling, and particle deposition on walls.
2. The observation of virus‐dependent inactivation results suggests that surrogate viruses for those causing infections in humans and animals (including pets and livestock) need to be selected for aerosol studies with strong consideration of virus structure. We suggest that future research should focus on identifying proper surrogate viruses. This will require identification of viruses which can be safely used in biosafety level 1 or 2 facilities, viruses which can be propagated in high titer (in excess of 10^7^ TCID_50_ ml^−1^) at high volume (10^2^ ml or more), and which behave similarly to their target virus in response to humidity changes, temperature changes,^1^, ^2^, ^51^, ^52^ oxidizing agents, visible and UV light exposure, and electric fields (ie, in response to variable scenarios encountered in the environment and in air pollution control technologies). Furthermore, future research is needed to better understand the particle size range where infectious viruses are encountered in aerosols in the environment. Not only does size affect particle deposition rates and deposition efficiency upon inhalation, but also control technologies for aerosolized viruses need to be evaluated considering challenge aerosols with the proper size distribution. A predominantly supermicrometer size distribution aerosol (by volume) was utilized in this study as currently available evidence suggests it is particles near one micrometer in diameter (but below 5 µm)^4^, ^17^, ^47^ which may carry both infectious SARS‐CoV‐2 and H1N1 influenza viruses.
3. Although wind tunnel testing can accurately yield removal efficiencies for particles, viruses, and gas phase pollutants, facilitating determination of clean air delivery rates for recirculating air purification technology, we must remark that the effectiveness of any recirculating air purification technology ultimately hinges upon the manner in which it is used. Technologies whose clean air delivery rate is small compared to the ventilation rate present in an indoor space will display a largely localized effect in terms of virus and particle removal, that is, an individual would need to stay close to the device in order to extract the benefits of the air purification unit. For this reason, while we argue wind tunnel testing is preferred to determine air purification unit penetration, collection efficiency, and correspondingly, clean air delivery rate, we also suggest that performance studies of such technologies in indoor environments (offices, residences, hospitals), akin to tests of how variable MERV rated filters ultimately influence indoor particle concentrations when installed in HVAC systems,^53^, ^54^, ^55^ need to be carried out.
4. In conjunction with the need to test recirculating units in representative test environments, changes in performance over time need to be assessed and device life time tests need to be carried out. The presented experiments in this study were limited to a relatively new test unit, and following virus aerosol trials, the unit was disposed of following Institutional Biosafety Committee protocol guidelines. Recirculating air purification units utilizing filtration will have time‐varying performance due to filter loading. While this can be rectified through regular filter replacement, temporal changes in pump/fan performance, as well as any components designed for particle electrostatic collection, virus inactivation, or oxidation (eg, electrostatic precipitators and UV‐sources) may change in performance over time.

## CONFLICT OF INTEREST

None of the authors have any financial or personal interests related to the results of this study.

## AUTHOR CONTRIBUTIONS

Yuechen Qiao was involved in data curation, formal analysis, investigation, validation, visualization, and writing the original draft, writing, reviewing and editing. My Yang was involved in conceptualization, data curation, formal analysis, investigation, and methodology, validation, visualization, writing the original draft, and writing, reviewing and editing. Ian A. Marabella was involved in investigation, methodology, validation, visualization, writing, reviewing, and editing. Devin A. J. McGee was involved in investigation, validation, and visualization. Bernard A. Olson was involved in conceptualization, funding acquisition, methodology, project administration, resources, supervision, writing the original draft, and writing–review and editing. Montserrat Torremorell was involved in conceptualization, funding acquisition, investigation, methodology, project administration, resources, supervision, writing the original draft, and writing, review and editing. Christopher J. Hogan Jr. ​was involved in conceptualization, data curation, formal analysis, funding acquisition, methodology, project administration, resources, supervision, visualization, writing the original draft, and writing, review and editing.

### PEER REVIEW

The peer review history for this article is available at https://publons.com/publon/10.1111/ina.12847.

## Supporting information

Supplementary Material

## Data Availability

Data have been provided as tables directly within the manuscript, and raw data are available via e‐mail upon request from the corresponding authors.
