# Supplementary material for: Wind tunnel‐based testing of a photoelectrochemical oxidative filter‐based air purification unit in coronavirus and influenza aerosol removal and inactivation
Source: Indoor Air. 2021 May 7;31(6):2058–69. doi: 10.1111/ina.12847 (PMC8242653; doi:10.1111/ina.12847)
Supplement: Supplementary file 1 — Supplementary Material [file INA-31-2058-s001.docx]

**Wind Tunnel Based Testing of a Photoelectrochemical Oxidative Filter-Based Air Purification Unit in Coronavirus and Influenza Aerosol Removal and Inactivation**

Yuechen Qiao^1**^, My Yang^2**^, Ian A. Marabella^1^, Devin A. J. McGee^1^, Bernard A. Olson^1*^_,_ Montserrat Torremorell ^2*^, Christopher J. Hogan Jr.^1*^

^1^Department of Mechanical Engineering, College of Science and Engineering, University of Minnesota, Minneapolis, MN 55455

^2^Department of Veterinary Population Medicine, College of Veterinary Medicine, University of Minnesota, Saint Paul, MN 55108, USA

**Supporting Information**

^**^Contributed equally to this work

^*^To whom correspondence should be addressed:

BAO: olso0209@umn.edu, 251 Mechanical Engineering, 111 Church St SE, Minneapolis MN, 55455

MT: torr0033@umn.edu, 335B Animal Science/Veterinary Medicine, 1988 Fitch Avenue, St. Paul, MN 55108

CJH: hogan108@umn.edu, 109 Mechanical Engineering, 111 Church St SE, Minneapolis MN, 55455

**Viral growth and Propagation & Cell Culture Testing Conditions**

*Bovine coronavirus*

Bovine coronavirus (NR-445, BEI Resources) was grown and titrated in HRT-18 cells. HRT-18 cells were maintained in Dulbecco’s Modified Eagle Medium (DMEM) (Life Technologies, Grand Island, NY, USA) supplemented with 10% fetal bovine serum (FBS, LifeTechnologies, Grand Island, NY, USA), and 1X antibiotic-antimycotic (Life Technologies, Grand Island, NY, USA). BCoV was propagated in HRT-18 monolayers between 80-90% confluency, and were maintained in BCoV growth media, composed of DMEM supplemented with 4% of 7.5% bovine serum albumin fraction V (Life Technologies, Grand Island, NY, USA), 1.5 µg/mL trypsin-TPCK (Sigma-Aldrich), and 1X antibiotic-antimycotic for 5 days at 37°C with 5% CO_2_. The virus was collected after two freeze-thaw cycles, centrifuged at 3,000 × g for 10 minutes, titrated, and frozen in 21 mL aliquots at -80°C until used in the experiment.

*Porcine respiratory coronavirus*

Porcine respiratory coronavirus (ATCC VR-2384) was grown and titrated in *Sus scrofa* testis (ST) cells as described in Qiao et al, 2020. Briefly, PRCV infected cells were maintained in PRCV growth media, composed of DMEM supplemented with 2% FBS and 1X antibiotic-antimycotic. PRCV infected cells were incubated for 2-3 days at 37°C with 5% CO_2_ until cytopathic effects (CPE) appeared. The virus was frozen at -80°C, thawed once, centrifuged at 3,000 × g for 10 minutes, titrated, aliquoted into 21 mL volumes, and frozen at -80°C until used in the experiment.

*Influenza A virus*

Influenza A virus was grown and titrated in Madin-Darby canine kidney (MDCK) cells. MDCK cells were maintained in Dulbecco’s Modified Eagle Medium (DMEM) supplemented with 10% fetal bovine serum (Life Technologies, Grand Island, NY, USA), 1X antibiotic-antimycotic (Life Technologies, Grand Island, NY, USA), 10 mM HEPES, and 1 mM sodium pyruvate. IAV was propagated in MDCK monolayers between 80-90% confluency. Infected cells were maintained in IAV growth media, composed of DMEM supplemented with 4% of 7.5% bovine serum albumin fraction V (Life Technologies, Grand Island, NY, USA), 1.5 µg/mL trypsin-TPCK (Sigma-Aldrich), 1X antibiotic-antimycotic, 50 µg/mL gentamicin (Lonza), and 50 µg/mL neomycin (Teknova) for 2 days at 37°C with 5% CO_2_. The virus was frozen at -80°C, thawed once, centrifuged at 3,000 × g for 10 minutes, titrated, aliquoted into 21 mL volumes, and frozen at -80°C until used in the experiment.

**Test KCL Particle Size Distribution Functions**


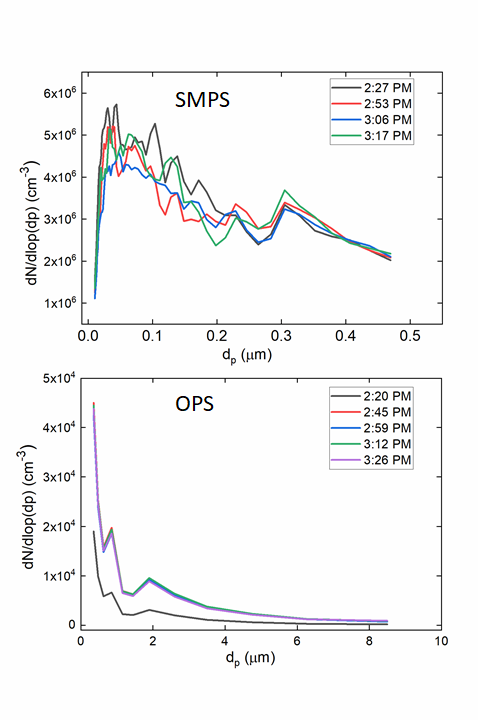


**Figure S1.** Upstream size distribution functions of particles inverted from SMPS (upper) and OPS (lower) measurements during particle penetration tests at a flow rate of 1416 L min^-1^. Penetration measurements follow ASHRAE guidelines. In the case of the SMPS measurements, the numerator for each of the four penetration values was the average of the two measurements bounding each of the upstream size distributions displayed, while the denominator was the displayed upstream size distribution function. For the OPS measurements, we applied the converse, and the numerator in each penetration calculation was one of the four distinct downstream size distributions, while each denominator was the average of consecutive upstream measurements. Upstream and downstream measurements were made in an alternating fashion in both instances.
